# Circumferential strain recovery after human cardiomyocyte transplantation in minipigs using a novel frequency-based method for myocardial tagging quantification

**DOI:** 10.1016/j.jocmr.2026.102756

**Published:** 2026-06-05

**Authors:** Anna V. Naumova, Kenta Nakamura, Silvia Marchiano, Lauren E. Neidig, Leslie P. Blakely, Hiroshi Tsuchida, Charles E. Murry, William S. Kerwin

**Affiliations:** a Department of Radiology, University of Washington, Seattle, Washington, USA; b Institute for Stem Cells and Regenerative Medicine, University of Washington, Seattle, Washington, USA; c Department of Medicine, Division of Cardiology, University of Washington, Seattle, Washington, USA; d Department of Laboratory Medicine and Pathology, University of Washington, Seattle, Washington, USA; e Center of Cardiovascular Biology, University of Washington, Seattle, Washington, USA; f Department of Comparative Medicine, University of Washington, Seattle, Washington, USA; g Currently at Fred Hutchinson Cancer Research Center, Seattle, Washington, USA; h Currently at Department of Stem Cell Biology and Regenerative Medicine, University of South California, Los Angeles, California, USA; i Currently at Altius Institute for Biomedical Sciences, Seattle, Washington, USA

**Keywords:** myocardial strain, quantitative measurements, large animal models, cell transplantation, myocardial infarction

## Abstract

**Background:**

Transplantation of human pluripotent stem cell-derived cardiomyocytes (hPSC-CMs) is a promising new method for heart remuscularization after infarction. We hypothesized that hPSC-CMs affect heart function by improving local contractility in the infarcted zones. However, there is a need for a precise noninvasive assessment of regional contractile function in the infarcted segments.

**Methods:**

We describe here a novel approach for rapid and robust quantification of myocardial end-systolic circumferential strain (CS). Linear tags are placed in 60-degree pattern offsets and analyzed via optimized post-processing based on local Fourier transformation of standardized American Heart Association (AHA) myocardial segmentation. This method has been implemented for the first time to evaluate transendocardial hPSC-CMs transplantation in a minipig model of myocardial infarction. Validation of the new frequency-based calculation of myocardial strain was done using two independent approaches as follows: (1) a tag tracking, and (2) feature tracking technique.

**Results:**

In the cell-treated hearts (n=4), mean end-systolic CS in the infarcted segments (anterior and anteroseptal areas combined) at the mid-wall region decreased from −6.69 ± 1.56% (pre-MI) to −1.13 ± 1.96% at 2 weeks post-MI (pre-treatment), with subsequent improvement to −4.00 ± 0.76% by 8 weeks after cell transplantation. Conversely, CS in the infarcted segments in vehicle-control group (n=5) decreased from −5.18 ± 0.97% (pre-MI) to −1.39 ± 1.23% at 2 weeks post-MI and worsened further to 0.33 ± 1.93% by 8 weeks post-vehicle. There was no improvement in the global ejection fraction in the cell-treated group in comparison with control. It was a high correlation of the new method of myocardial strain calculation with the standard tag tracking approach and feature tracking strain analysis across the experimental conditions (normal heart, infarcted, cell/vehicle treated).

**Conclusions:**

A novel frequency-based technique for assessment of local circumferential strain does not require specialized acquisition protocols, access to k-space data, nor highly optimized reconstruction algorithms or commercial software. It can quickly and precisely assess regional myocardial injury and recovery. Our findings support our hypothesis that transplantation of hPSC-CMs improves regional myocardial strain in infarcted minipig hearts.

## Graphical abstract

**Figure:**
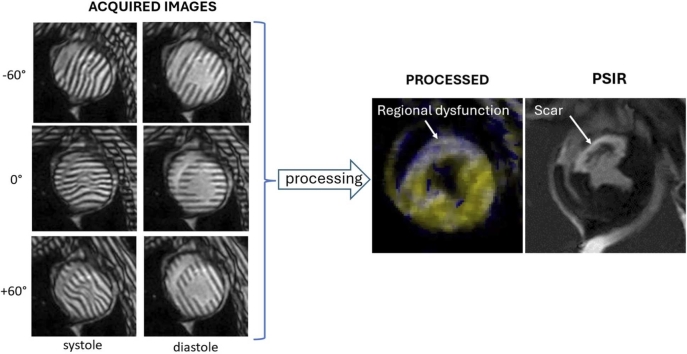

## 1 Background

Regenerative therapies represent the new frontier for treating myocardial infarction. Recent studies have shown that intramyocardial delivery of human pluripotent stem cell-derived cardiomyocytes (hPSC-CMs, either embryonic, hESC-, or induced-pluripotent, hiPSC-) improved global left ventricle (LV) ejection fraction and decrease adverse remodeling after myocardial infarction (MI) in infarcted non-human primates (NHP) [1], [2], [3], [4]. Shiba et al. [2] reported improvement in fractional shortening of the anterior LV wall after hESC-CM transplantation in NHP. In larger animals such as pigs, measures of global and regional contractility (ejection fraction and fractional shortening) have not shown consistent improvement [5], [6]. A novel measure of contractility sensitive to cardiac cell therapy is thus needed.

Cardiovascular magnetic resonance (CMR), indeed, is the gold standard for noninvasive assessment of heart geometry, global and regional contractility [7], [8]. Myocardial strain, defined as changes in regional myocardial deformation, can identify subclinical myocardial dysfunction and predict cardiovascular events [9], [10], [11], [12]. We hypothesized that the assessment of myocardial strain after cardiac cell therapy may provide a better assessment of the efficacy of this novel treatment than global ejection fraction.

Despite the development of newer imaging methods of myocardial strain assessment, such as CMR feature tracking [12], [13], [14], [15] and deep learning synthetic strain [16], the classic reference technique for myocardial strain quantification is CMR tagging [7], [11], [17], [18], [19], [20]. Tagging requires a dedicated acquisition where a grid is generated with an RF/gradient pulse sequence followed by a strong gradient saturation pulse. Moreover, post-processing of the tagging data is challenging and is not available in standard clinical MRI software. Indeed, the quantification of tagging grid deformation is usually obtained by laborious post-processing and custom-written software not available for routine clinical use. For example, harmonic phase analysis (HARP) allows tissue points tracking through time and calculation of circumferential and radial Lagrangian strain from the points tracked in k-space [21], [22]. Other more sensitive, but still challenging, approaches characterized by low signal-to-noise ratios are Displacement ENcoding with Stimulated Echoes (DENSE) that quantifies tissue motion into the image phase with encoding and decoding gradients [23], and Strain ENCoding (SENC) that relies on pixel intensity [24]. While effective for calculating myocardial strain, these techniques also are laborious and require dedicated acquisitions and specialized post-processing on custom-written software. There is a need for a simplified quantification method that is easily accessible and applicable across various settings.

The aim of the study was to (1) develop a rapid and accurate method for assessment of regional myocardial deformation from tagging images; (2) validate the new method with standard tag tracking and feature tracking techniques; and (3) test the robustness of this technique to assess efficacy following transendocardial cardiac cell therapy in a minipig model of myocardial infarction.

To facilitate strain analysis, we developed a new strategy for placing and analyzing tag patterns, where 3 separate orientations of parallel tag lines were placed such that one set of tags was approximately perpendicular to the myocardial wall in the 6 segments according to standard American Heart Association (AHA) segmentation nomenclature. Local strain was then evaluated by measuring the change in frequency of the local tag pattern during contraction. We used this approach to test whether transplantation of hPSC-CMs improves regional myocardial strain in the infarcted zone of the minipig’s heart.

## 2 Methods

### 2.1 Animals

Nine Yucatán minipigs (all males, 30–40 kg) were included in the study. All procedures were approved and conducted in accordance with the University of Washington (UW), the Office of Animal Welfare and the Institutional Animal Care and Use Committee (IACUC). Animals were housed in the facilities of the UW Department of Comparative Medicine in compliance with the principles of the Guide for Laboratory Animal Facilities and Care of the National Academy of Sciences, National Research Council. The minipigs were monitored at least twice a day and were under the care of UW staff veterinarians with extensive experience in large animal health and in consultation with clinical cardiologists. Animals received ad libitum water and were fed twice a day (Lab Diet – Laboratory Porcine Growth Diet 5081 and 5K99, LabDiet, St. Louis, Missouri). Animals have a minimum of 5-day acclimation period before being enrolled in the study.

### 2.2 Myocardial infarction

Myocardial infarction (MI) was created using a closed-chest technique of myocardial ischemia followed by reperfusion [25]. Briefly, the femoral artery was accessed by surgical exposure and coronary angioplasty balloon (Boston Scientific, Marlborough, Massachusetts) was positioned and inflated in the mid left anterior descending artery (LAD, distal to the first large diagonal branch) to induce ischemia for 90 min followed by reperfusion under fluoroscopic guidance. MI was confirmed by coronary angiography and ST-segment elevation on electrocardiogram (ECG). During the infarct procedure, a central jugular venous catheter (Access Technologies, Skokie, Illinois) and telemetry unit (EMKA easyTEL+, Emka Technologies, Paris, France) were placed and maintained until the animal reached endpoint. For all surgical procedures, animals were induced with a combination of butorphanol, acepromazine and ketamine administered intramuscularly. Animals were intubated and mechanically ventilated using isoflurane and oxygen to maintain a surgical plane of anesthesia. Vital signs were measured continuously throughout the procedure. All anesthetic and surgical procedures are performed under the care and supervision of a veterinarian. All animals received Buprenorphine SR-Lab (ZooPharm, Laramie, Wyoming) for post-operative analgesia after surgical procedures.

### 2.3 Preparation of human pluripotent stem cell derived cardiomyocytes (hPSC-CM) for intramyocardial injection

Human embryonic stem cells RUES2e002-A (Rockefeller University, New York, New York) were maintained and differentiated into cardiomyocytes as previously described [25], [26]. All pigs were immunosuppressed with methyl prednisolone and abatacept as previously described [25]. Briefly, hPSCs were adapted to suspension cultures using Essential 8 complete media (Gibco, Waltham, Massachusetts). After expansion, hPSC were treated with an optimized concentration of CHIR99021 (Cayman Chemical, Ann Arbor, Michigan) for 48 h followed by WNT inhibition. Cardiomyocyte state was reached 6 days after CHIR induction and non-cardiac cells were eliminated from the cultures by purification with sodium lactate (4 mM, Sigma-Aldrich, Burlington, Massachusetts). Until the day of the harvest (day 20–22), hPSC-CMs were maintained using RPMI-1640, supplemented with B27 (Gibco). 24 hours before harvesting, hPSC-CMs were heat-shocked at 42°C for 1 h; the next day, the hPSC-CMs were dissociated using TrypLE (Gibco) and cryopreserved using Cryostore (Sigma-Aldrich). On the day of injection, hPSC-CMs were quickly thawed in the water bath (37°C) and diluted in RPMI-1640, without phenol red to reach the concentration of 0.5×10^6^ hPSC-CMs/μL. Cells were then loaded into 1cc syringes (BD) and sterile-packed on ice until injection.

### 2.4 hPSC-CM transplantation

Cell transplantation was performed two weeks after infarct by transendocardial injection into the peri-infarction region using the NOGA-MyoStar endocardial mapping and delivery system (BioSense Webster, Irvine, California) as previously described with minor modification [25]. Briefly, the infarct region was first mapped using the NOGA catheter, and then the MyoStar catheter to deliver 15–discrete transendocardial injections of 100 µl each for a total dose of 5×10^8^ hPSC-CMs. Injections were only performed with excellent location and loop stability, ST-segment elevation and presence of premature ventricular contraction with needle insertion in an appropriate location by electroanatomical map and unipolar volage. Two-thirds of injections were placed into the peri-infarct border zone defined by unipolar voltage of 5–7.5 mV and the remaining one-third into the central infarct region defined as unipolar voltage of <5 mV.

### 2.5 Cardiovascular magnetic resonance (CMR)

*In vivo* imaging studies were conducted on a 3T Ingenia CX clinical scanner (Philips Healthcare, Best, the Netherlands) on healthy animals at one-two days before MI modeling, then at 2 weeks after MI, and again at 4 and 8 weeks after cell or vehicle injection. During the CMR scan, the animals were sedated with a combination of butorphanol, acepromazine and ketamine administered intramuscularly. Animals were then intubated and mechanically ventilated using isoflurane and oxygen to maintain anesthesia during the scan.

**Fig. 1:**
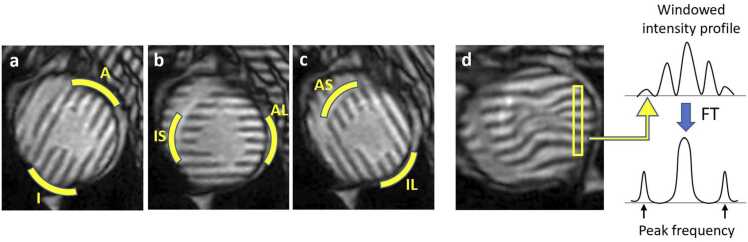
Placement of the tagging lines in the short axis of the heart tangential to the heart wall with 60-degree shift to each other. Panels **a**, **b**, and **c** show alignment of the zones of circumferential sensitivity with the standard AHA segments labeled. Tagged images in the panels **a–c** are shown in the end-diastolic phase of cardiac cycle. Panel **d** shows a scheme of frequency assessment by local Fourier transformation in the zones of circumferential sensitivity. The tagged image in panel **d** is shown at the end-systolic phase of cardiac cycle. AHA segments are labeled as following: anterior (A); anteroseptal (AS); inferoseptal (IS); inferior (I); inferolateral (IL); anterolateral (AL). An additional cine images in the PowerPoint file show this in more detail [Additional_file 11] *AHA* American Heart Association

The CMR protocol included a balanced turbo field echo (bTFE) cine sequence for assessment of the heart contractility; complimentary spatial modulation of magnetization (CSPAMM) sequence was used for tagging imaging; late gadolinium enhancement scan with phase-sensitive inversion recovery (PSIR) pulse sequence was used for measuring the infarct size [27]. The tagged acquisition was done on a single short-axis slice placed between mid-ventricle and apex of the heart through the infarcted zone (slice 4 from the apex). This allowed us to get strain data from the infarcted zone, borderline, and opposite to infarcted segments of the heart. Tagging lines were placed tangentially to the heart wall with 60-degree shift to each other and were aligned with the standard AHA segments [28] (Fig. 1a-c). The parameters of the CSPAMM sequence were the following: TR 5.8 ms; TE 3.5 ms; flip angle (FA) 10 degree; field of view 350×350 mm; slice thickness 8 mm; image resolution 1.1×1.1 mm; 3 mm tag separation. All acquisitions were done with 1 signal average, ECG-gated and with breath hold. Tagging image acquisition time was less than one minute per three acquisition planes (60-degree shift to each other). CSPAMM acquisition was done after cine multislice before injection of gadolinium.

For infarct visualization, minipigs subsequently received an intravenous injection of gadolinium (Gd)-based contrast agent ProHance (0.2 mmol/kg, Bracco Diagnostics Inc., Princeton, New Jersey) bolus followed by a saline flush. Delayed contrast-enhanced images were acquired at the short axis of the heart with an ECG-gated, breath-hold PSIR sequence acquired at 10 min after the contrast agent injection. The inversion time was adjusted by the scanner operator after the look-locker acquisition to null signal from non-infarcted remote myocardium. PSIR acquisition parameters included TR of 7.1 ms, TE of 3.5 ms, FA of 25°, inversion time (TI) range 280–350 ms, field of view 250×250 mm, slice thickness was 6 mm without gap between slices, one signal average. All PSIR images were acquired at the end-diastolic phase of cardiac cycle.

### 2.6 Tagging analysis

A new image processing technique for rapid analysis of tagged CMR images was implemented in MATLAB (Mathworks, Natick, Massachusetts). Similar to HARP [19], changes in the spatial frequency of the tagged pattern due to heart motion were used to determine strain, with the exception that our technique did not require specialized acquisition protocols, access to k-space data, nor highly optimized reconstruction algorithms. Instead, we employed localized Fourier transforms perpendicular to the applied tag pattern to measure changes in peak frequency during the cardiac cycle. By using multiple tag orientations and limiting the analysis to circumferential strain, we ensured several cycles of the tag pattern could be sampled within the myocardium for robust frequency estimation.

For each pixel at each time frame and tag orientation, image intensities were sampled perpendicular to the tag line orientation. The intensity profile was then multiplied by a Gaussian window with a standard deviation of 4 pixels and the result underwent Fourier transformation (Fig. 1). The peak spatial frequency was identified and used to determine perpendicular strain at the pixel using

*e*_perpendicular_ = (*f*_peak_-*f*_0_)/*f*_0_

where *f*_0_ is the applied tag frequency. Essentially, this uses the inverse relationship between the relative fiber length and apparent tag pattern frequency (e.g., shortening by 50% doubles the tag frequency).

To obtain measurements of circumferential strain, a user-selected point at the center of the LV is identified and a frame identifying the 6 AHA segments is aligned with the center of the myocardial wall. The circumferential strains for pixels within each AHA segment are computed by first determining the tag orientation closest to the line connecting the center of the LV to the pixel. The angle θ between the connecting line and the tag orientation is used to account for the local orientation of the LV wall giving

*e*_circumferential_ = *e*_perpendicular_ / cos(θ).

For values of θ between 20 and 40 degrees, a weighted average with the adjacent tag orientation is used to ensure continuity of measurements. For θ greater than 40 degrees, other tag orientations are used. Finally, the average circumferential strain within each AHA segment is recorded. Resulted circumferential strain (CS) data are shown as negative numbers that correspond to myocardial shortening (contraction); positive strain corresponds to lengthening, whereas values of increasing strain (toward positive values) reflect worsening contractile function in that region. Less negative strain values represent hypokinetic myocardium. A value of 0 represents akinetic non-contractile myocardium, and a positive value represents dyskinetic myocardial segments. Global peak CS reflects the average of maximal strain values in six AHA segments over the circumference of the LV. The regional end-systolic strain measurements in 6 AHA segments represent the circumferential strain in the end-systolic phase of cardiac cycle. Strain rate (SR) was calculated as the amount of strain accumulated from the first to third phase of the heart cycle and expressed in %/s.

### 2.7 Method validation

Validation of the new frequency-based calculation of myocardial strain was done using two independent approaches: (1) a tag tracking approach, and (2) feature tracking technique. Tag tracking forms the foundation of established methods such as FindTags [29] that has validated the HARP technique [21]. The fractional change in distance between tag lines provides a measurement of strain perpendicular to the lines. Because our method measures circumferential strain, we identified locations where the tag patterns were perpendicular to the circumference of the LV myocardium and measured the fractional change in distance between tag features to estimate circumferential strain. The distance between tag features was measured for each phase by identifying the two nearest peaks and valleys in intensity in each direction after zero-filled Fourier interpolation. The average separation between these points was then measured and the implied strain was compared to corresponding values from our method.

Feature tracking (FT) analysis was performed using Circle Cardiovascular Imaging software (CVI42, Circle Cardiovascular Imaging Inc., Calgary, Alberta, Canada) to measure myocardial strain and strain rate from short-axis bTFE cine multislice images. FT-based circumferential strain results specific to the 4th from apical slice were recorded to match the slice position with CSPAMM-tagged acquisition from the same slice.

### 2.8 Statistical analysis

Statistical analyses were carried out in Excel software for Windows (Microsoft Inc., Redmond, Washington ,). Results are presented as mean ± standard error of mean (SEM). Differences in myocardial strain and strain rate between cell- and vehicle-treated animals were tested using t-test for independent samples. Differences in strain assessed in different study points were tested using paired t-test. Differences were considered statistically significant with p values less than 0.05. Coefficient of variation between repeated measurements was assessed using the root mean square method [30]. Pearson correlation was used to evaluate correspondence between strain results obtained by different methods.

## 3 Results

### 3.1 Left ventricular strain pattern in healthy minipig heart

**Fig. 2:**
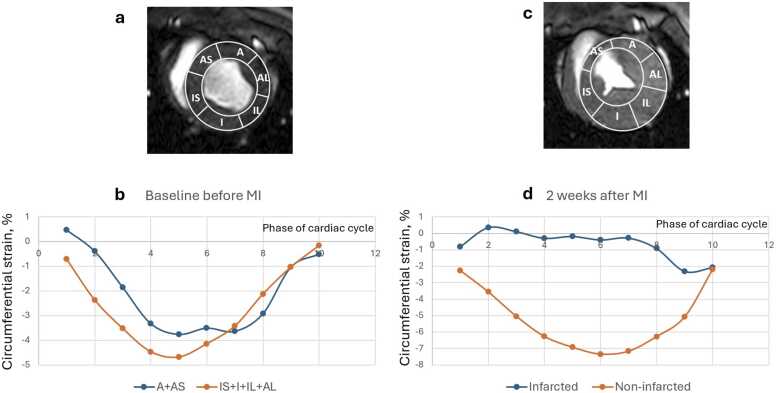
The temporal dynamics of the mean circumferential strain in normal (baseline, n=9) and infarcted (n=9) minipig’s heart over the 10 phases of cardiac cycle shown for the infarcted (anterior and anteroseptal segments combined) and non-infarcted (inferoseptal, inferior, inferolateral, anterolateral segments combined) zones of left ventricle. (a) AHA segment location in the normal heart. (b) Dynamic changes of circumferential strain (CS) in the combined anterior and anteroseptal segments (A+AS) and combined inferoseptal, inferior, inferolateral, anterolateral (IS+I+IL+AL) segments in the normal heart over cardiac cycle. (c) AHA segment location in the infarcted heart. (d) Dynamic changes of the CS in the infarcted (anterior and anteroseptal segments combined) and non-infarcted (inferoseptal, inferior, inferolateral, anterolateral combined) segments over cardiac cycle in two weeks after MI. AHA segments are labeled as follows: anterior (A); anteroseptal (AS); inferoseptal (IS); inferior (I); inferolateral (IL); anterolateral (AL). *AHA* American Heart Associa

Circumferential strain (CS) and strain rate (SR) first were measured in the normal heart before myocardial infarction (baseline). Global mean peak CS of the minipig’s heart was −5.95 ± 0.85% (mean ± SEM, n=9). Regional mean end-systolic CS in six AHA segments in the circumference of the heart varied from −5.22 ± 0.84% (inferoseptal segment) to −8.00 ± 1.18% (inferior segment) in the healthy myocardium. Per-segment strain results are shown in the supplemental file (Additional file 1). Global mean peak SR of the normal heart was −30.8 ± 3.2%/s. There were also modest regional variations of the SR between segments of the left ventricle (LV) wall, but the differences were not statistically significant (Additional file 1). Fig. 2 shows dynamic changes of the regional CS in the combined anterior and anteroseptal segments (A+AS) and combined inferoseptal, inferior, inferolateral, anterolateral (IS+I+IL+AL) segments in the normal heart during myocardial contraction. Strain increased from the end-diastolic phase reaching the peak at the end-systolic phase (phases 4–5) and decreases to diastolic values by phase 10. All segments of the normal minipig’s heart had similar patterns of CS changes during cardiac cycle (Fig. 2).

### 3.2 Left ventricular strain after myocardial infarction (MI)

**Fig. 3:**
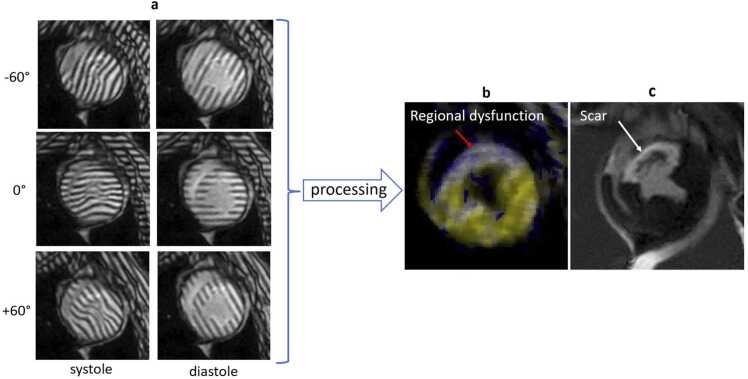
A new myocardial tagging method reveals the area of regional myocardial dysfunction in the minipig’s heart that corresponds to the infarcted zone on PSIR images. (a) Acquired tagged images in mid-ventricular level of the infarcted minipig’s heart shown at the end-diastolic and end-systolic phases of cardiac cycle. (b) Processed tagged images reveal area of regional dysfunction (red arrow) located at the anterior and anteroseptal segments (yellow indicates shortening and blue, lengthening). (c) PSIR image of the same mid-ventricular slice of the heart acquired at 10 min after iv injection of gadolinium (0.2 mmol/kg) reveals zone of scar as hyper-enhanced area of LV located at the anterior and anteroseptal segments. *PSIR* phase-sensitive inversion recovery

MI caused significant LV remodeling (increase in LV chamber volume and mass), a decrease in ejection fraction in 2 weeks after injury (Additional file 9), and a decrease in CS and SR in the affected heart segments (anterior and anteroseptal AHA segments, Table 1). Reductions in CS and SR were reflected in more positive values after MI. Regional changes in the CS and SR of each AHA segment are detailed in Additional files 1–8. While global peak CS at 2 weeks after MI did not show any difference compared to pre-infarcted baseline values, the regional decrease in end-systolic CS was significant for the infarcted anteroseptal segments (anterior and anteroseptal combined) of the heart 2 weeks after infarction from −5.83 ± 0.69% (baseline, pre-MI) to −1.28 ± 1.07% (2 weeks post-MI, p = 0.0007). There were no changes in CS in non-infarcted (inferoseptal, inferior, inferolateral, anterolateral combined) segments of the LV (Table 1). Location of dysfunctional LV areas calculated with the new tagging algorithm well matched with the infarction zone enhanced by gadolinium in PSIR images (Fig. 3). Myocardial infarction affected systolic strain rate (SR) of the minipig’s heart. Global SR after MI changed from the normal baseline values of −30.80 ± 3.25%/s to −21.13 ± 2.60%/s at 2 weeks post-MI (p = 0.0105). The regional differences in SR between normal and infarcted hearts were also statistically significant in the infarcted segments (anterior and anteroseptal combined) of the minipig’s heart (p = 0.0004, Table 1). The AS segment was mostly affected by MI, and its dyskinesia is reflected by positive values of CS and SR in that area (Additional files 2 and 6).

**Table 1:** Global and regional peak systolic circumferential myocardial strain (CS) and strain rate (SR) in normal minipig’s heart (baseline) and at 2 weeks after myocardial infarction (MI) (n=9) assessed with novel frequency-based and standard feature-tracking methods

		Frequency-based method				Feature-tracking method		
	Measurement, units	Global	Infarcted segments	Non-infarcted segments	Measurement, units	Global	Infarcted segments	Non-infarcted segments
Before MI	CS, %	−5.95±0.85	−5.83±0.69	−6.59±0.51	CS, %	−13.73±2.07	−13.27±1.19	−14.08±1.10
	SR, %/s	−30.80±3.25	−28.00±3.67	−32.20±3.14	SR, s^−1^	−1.23±0.09	−1.24±0.45	−1.30±0.33
After MI	CS, %	−6.02±0.43	−1.28±1.07 #	−7.68±0.67	CS, %	−10.26±0.72	−1.08±2.62 #	−12.81±1.37
	SR, %/s	−21.13±2.60 #	−0.87±6.15 #	−26.26±3.40	SR, s^−1^	−0.66±0.06 #	1.81±1.55 #	−1.48±0.46

Results of 6 AHA segments are combined to infarcted (anterior and anteroseptal segments combined) and non-infarcted (inferoseptal, inferior, inferolateral, anterolateral segments combined) zones of left ventricle. Results are shown as mean ± SEM. Statistically significant differences between normal (baseline, before MI) and infarcted (2 weeks after MI) values are marked with **# (p < 0.05).***SEM* standard error of the mean, *AHA* American Heart Association

Regional decrease in CS and SR at two weeks after MI was also detected with the feature tracking (FT) method as shown for the infarcted and non-infarcted segments (Table 2) and for each of 6 AHA segments (Additional file 2). The decrease in strain and strain rate was statistically significant in the infarcted segments in comparison with the baseline pre-MI as calculated by both methods.

**Table 2:** Coefficients of variation (CV) between repeated measurements of circumferential strain (CS) and strain rate (SR) in the infarcted (anterior and anteroseptal segments combined) and non-infarcted (inferoseptal, inferior, inferolateral, anterolateral segments combined) segments of the minipig’s heart measured by the novel frequency-based and standard feature-tracking methods

	Frequency-based method			Feature-tracking method		
	Global	Infarcted segments	Non-infarcted segments	Global	Infarcted segments	Non-infarcted segments
CV CS, %	3.6	3.0	6.2	13.0	26.5	20.3
CV SR, %	4.8	7.5	6.0	8.7	19.1	22.0

Temporal dynamics of mean regional CS in the infarcted and non-infarcted segments during myocardial contraction in the infarcted heart are shown in Fig. 2. The lack of contractility (or impaired circumferential shortening) of the infarcted area (anterior and anteroseptal segments combined) of the left ventricle can be characterized as stretching or lengthening of the scar, the non-infarcted lateral LV wall instead demonstrated a compensatory increase in CS (Fig. 2). In comparison with the pre-infarcted values, circumferential strain in the non-infarcted segments increased by 30%–45%, which might be related to compensatory hypertrophy and hyperfunction.

### 3.3 Left ventricular strain and strain rate after hPSC-CM transplantation

**Fig. 4:**
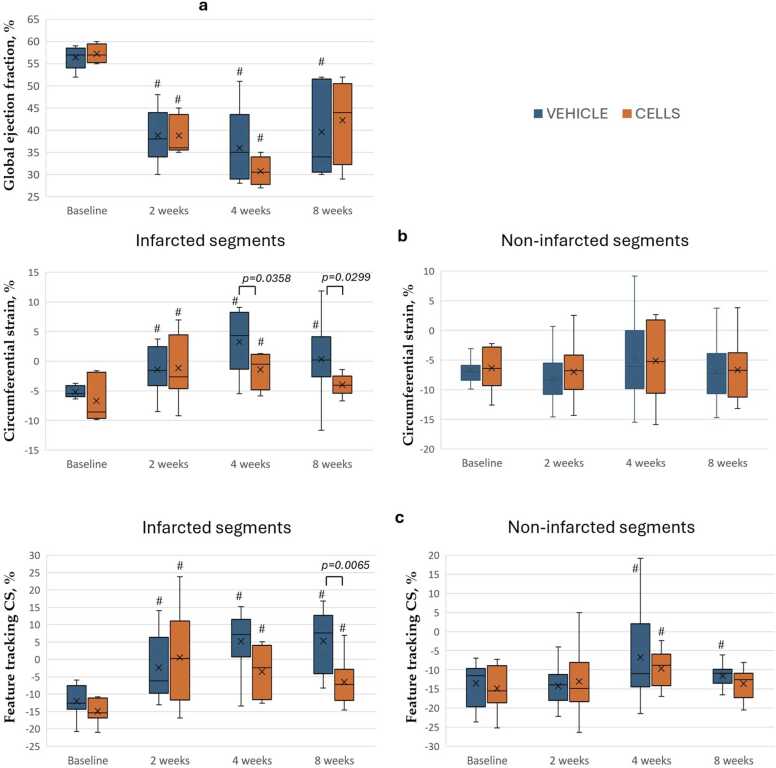
Longitudinal changes of the global ejection fraction (EF) (a), mean circumferential peak strain calculated using novel frequency-based approach (b), and mean circumferential peak strain calculated using standard feature tracking method (c) in the infarcted region (anterior and anteroseptal segments combined) and non-infarcted LV region (inferoseptal, inferior, inferolateral, anterolateral segments combined) of the minipig’s heart following cardiac cell therapy or vehicle injection. # marks statistically significant difference with the baseline values of each studied group (p<0.05). Excel-built box plots display data as follows: minimum (bottom whisker end), first quartile (bottom box edge), median (line in box), third quartile (top box edge), and maximum (top whisker end). “x” marks the mean value. There were no differences between the control and cell-treated cohorts in global EF. Regional CS significantly improved at 4- and 8-weeks post-treatment with hPSC-CM transplantation into the anterior/anteroseptal infarct territories (orange, n=4) compared to vehicle-control group (blue, n=5). No differences were observed in the non-infarcted (inferoseptal, inferior, inferolateral, anterolateral) regions. *LV* left ventricular, *hPSC-CM* human pluripotent stem cell cardiomyocytes.

The parameters of the global contractile function of the infarcted minipig’s heart, such as ejection fraction, end-systolic and end-diastolic volumes, and stroke volume, were not statistically different between the control and cell-treated group in any of the studied time-points, except of the larger ESV at 4-weeks in cell-treated animals (Fig. 4a and Additional file 9). In contrast, regional myocardial end-systolic strain and strain rate successfully detected beneficial effects of hPSC-CM transplantation in the infarcted zone (anterior and anteroseptal LV segments). Specifically, transendocardial intramyocardial delivery of hPSC-CMs to the infarcted LV areas significantly increased the end-systolic CS in the infarcted segments of the heart (Fig. 4b) at 4- and 8 weeks after transplantation, while infarcted areas remained dyskinetic/akinetic in control animals after MI for the duration of the study. In cell treated hearts, mean end-systolic CS in the infarcted LV segments (anterior and anteroseptal segments combined) at the mid-wall region decreased from −6.69 ± 1.56% (pre-MI) to −1.13 ± 1.96% at 2 weeks post-MI (p = 0.0234, difference to pre-MI, paired t-test), with subsequent improvement to −4.00 ± 0.76% by 8 weeks after cell transplantation (p = 0.0821, no difference to pre-MI, paired t-test). Statistically significant differences in myocardial end-systolic strain in the infarcted segments between vehicle-control and cell-treated groups were found in the 4 weeks (p=0.0358) and 8 weeks (p = 0.0298) after cell/vehicle administration (Fig. 4b and Additional files 1–4). This improvement in regional function in the infarcted area of the cell-treated animals was associated with viable graft in this region, as confirmed by histological analysis shown in Additional file 10. There was a general trend toward compensatory increases of CS in the non-infarcted segments (inferoseptal, inferior, inferolateral, anterolateral segments combined), although only the anterolateral segment at 8 weeks achieved statistical significance (p = 0.0249, Additional file 8). Representative tagged cine images of the vehicle control and cell-treated hearts show changes in regional contractility over time (Additional file 11).

hPSC-CM transplantation caused a significant increase in the regional myocardial strain rate of the infarcted LV segments at 4- and 8-weeks post-transplantation with subsequent recovery at 8-weeks almost to pre-infarcted values in the cell-treated cohort but did not in the vehicle-control (Additional files 7 and 8).

To substantiate the performance of the new frequency-based approach, we demonstrated similar directions in circumferential strain changes over time using feature tracking (FT) strain analysis across the experimental conditions (normal heart, infarcted, cell/vehicle treated). Despite some difference in absolute values of myocardial strain calculated by different methods, statistically significant improvements in circumferential strain after cell transplantation to the infarcted zone were recorded by both methods by week 8 post-treatment (Fig. 4). However, circumferential strain results obtained with feature tracking method had larger variability, and, despite of the similar trend, regional CS differences between cell-treated and vehicle-control groups in the infarcted zone (anterior and anteroseptal segments combined) did not reach statistical significance at 4-week post-treatment (Fig. 4c), while our novel frequency-based approach detected the difference in strain at 4- as well as at 8-weeks post-treatment (Fig. 4b).

### 3.4 Method validation

**Fig. 5:**
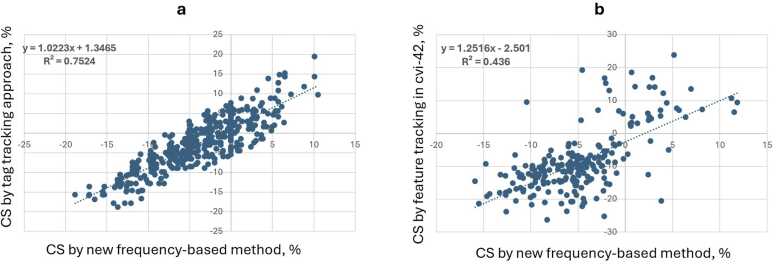
Validation of a new frequency-based calculation of myocardial circumferential strain in comparison to classical tag tracking technique (a) and comparison to feature tracking method (b) in all segments and all studied time points (n=9). *CS* circumferential strain

The accuracy of a new frequency-based calculation of myocardial strain in comparison to the established tag tracking approach is shown in Fig. 5a. The correlation coefficient between CS values obtained with two methods was 0.86785.

Myocardial FT-based analysis of bTFE cine multislice images was performed in CVI42 software at the baseline, 2-weeks post-infarction, and 4 and 8 weeks after cell or vehicle injection. There was a strong correlation (Pearson’s correlation coefficient 0.66033) between myocardial circumferential strain results obtained with our new frequency-based and FT-based methods (Fig. 5b). The per-segment results of the myocardial circumferential strain assessment with feature tracking method are included in Additional files 1–8.

The new frequency-based method of myocardial circumferential strain and strain rate calculation is highly reproducible as shown by low coefficients of variation (CV) between repeated measurements (Table 2). Feature tracking CS and SR repeated calculation results were characterized by higher variability as shown by higher CV values (Table 2).

## 4 Discussion

Myocardial tissue tagging as a magnetic resonance-based noninvasive imaging method for tracking myocardial motion was first introduced by Zerhouni et al. [18] and has been further developed over the past 30 years. The number of important milestone techniques includes HARP, DENSE, SENC, FT, but all those methods require either complicated post-processing, or capability of working with data in k-space, or might be laborious and time-consuming, often expensive, required custom-written patent-protected software not available for routinely for research and clinical applications. There is a need for development of more precise and/or simpler approaches for fast and reliable quantification of myocardial strain. One area of development might be related to analysis of tagged images. The analysis methods vary from simple visual inspection of tag deformation to exhaustive calculations of different strain components. The basic idea behind all tagging analysis techniques is tracking adjacent tag intersection points and measuring relative increases or decreases of their distances from one time frame to another to calculate strain.

In this study, we demonstrate a simple approach for rapid and robust quantification of myocardial circumferential strain. By placing linear tags in 60-degree pattern offsets aligned with AHA segmentation and processed using local Fourier transformation, we provide an accessible method for optimized segmental analysis. To demonstrate the robustness of this method to assess clinical response, we evaluate the global and regional myocardial circumferential strain in a pilot minipig study of cardiac cell therapy. Mid-wall LV circumferential strain is the most frequently computed parameter for quantifying regional myocardial function. This particular strain measure is favored and less susceptible to noise due to the abundance of tagging data around the mid-wall myocardial circumference [31], [32]. Circumferential strain is also a sensitive index of myocardial recovery compared with traditional measurements of LV contractility such as ejection fraction.

The key advantages of our new approach are the following:

- Fast acquisition based on CSPAMM sequence.
- 60-degree tag pattern that allows precise quantification of circumferential strain in each of 6 AHA segments.
- Optimized post-processing based on local Fourier transformation of the defined AHA segments.
- Quick image processing in MATLAB that produces circumferential strain maps.

In comparison with other known CMR methods for myocardial strain assessment, such as HARP, DENSE, SENC, and FT, our novel frequency-based approach does not require time-consuming complicated post-processing, k-space analysis, obtaining licenses to a patent-protected commercial software. Our newly developed frequency-based method for regional analysis of tagged images can be easily programmed using any available mathematical software, such as MATLAB. The new method has the potential to streamline analysis if implemented within radiology PACS environment because it consists of a mathematical algorithm applied to the images without complicated or time-consuming post-processing. Our novel method is fast and simple in use, it can be easily applied to variety of research and clinical applications and different myocardial conditions (normal, pathology, regeneration).

The new frequency-based method of myocardial circumferential strain analysis was applied to study changes in global and regional strain and strain rate of infarcted Yucatán minipigs and their response to cardiac cell therapy with intramyocardial transplantation of hPSC-CMs. In our study, the global mean peak CS of the healthy minipig’s heart was estimated as −5.95 ± 0.85% and consistent with the CS previously reported for normal Yorkshire farm pigs (−6.5 ± 1.3%) [33]. Regional mean peak strain was similar in all AHA segments in the circumference of the heart which is expected for the normal healthy myocardium. Higher peak circumferential strain results were reported in studies of human heart, such as −17.0 ± 2.4% when assessed with HARP method [9], −18.8 ± 1.6% when assessed with DENSE method [34], −17.9 ± 2.9% with SENC method [35], −16.0 ± 2.7% when assessed with the feature tracking method [36]. The differences in strain results might be related to a strain estimation method and slice position: in our study, tagged acquisition was done on a single slice through the infarcted zone located between mid-ventricle and apex of the heart. Another difference might be related to the image processing method. HARP method of tagging analysis has a disadvantage of increasing noise during diastolic frames due to T_1_ relaxation of the tagged myocardium and low spatial (8 mm distance between tags) and temporal resolution (∼ 50 ms) [37], [38]. Our method uses higher spatial (3 mm tag separation with 6 mm slice thickness) and temporal resolution that enables more precise measurements of the regional CS. Our new approach is faster and less noisy than other methods of tagging analysis, such as HARP that directly use k-space to estimate “instantaneous” frequency [21]. However, due to a very limited number of published tagging studies in normal pig hearts, the direct comparison of strain differences between species is challenging.

Myocardial infarction causes significant changes in the global and regional contractile function of the heart represented by increase in LV chamber volume, decrease in ejection fraction, and decrease in regional myocardial strain at two weeks after MI. The regional changes in strain and strain rate should be monitored closely, especially in the infarcted zone, to detect the local injury and subsequent treatment response. Based on our data, global average peak myocardial CS did not change significantly for 2 weeks after MI and decreased gradually by 4 and 8 weeks after vehicle administration. We observed a consistent trend for decreased global peak strain through all studied time points that was however not statistically significant. The regional end-systolic CS decrease in the injured hypokinetic segments of LV (infarcted anterior and anteroseptal segments) was more pronounced and reached statistical significance by 2 weeks post-MI with subsequent worsening at 4- and 8-weeks post-vehicle injection. Location of dysfunctional LV areas calculated using the new tagging method and visualized in MATLAB matched the infarction zone enhanced by gadolinium in PSIR images. This demonstrates the precision of our tagging method to detect dyskinetic areas of LV. Other studies also reported a decrease of circumferential strain in the infarcted zones of the pig heart. For example, CS was found to be −5.5 ± 0.3% [39], −2.5 ± 4.1% [40], and even to −1% in fully akinetic areas [38]. Our data showed increased CS of the remote areas, such as anterolateral and inferolateral segments, which might be related to the compensatory inotropy and hypertrophy. This increase in CS in the segments opposite the infarct territory segments has been described previously; mean CS −18% was reported in the remote areas of the infarcted minipig’s myocardium [38]; −10–15% [39], and −20.1 ± 5.1% [40].

Transendocardial delivery of hPSC-CMs improved global and regional peak circumferential strain, especially in the targeted infarct segments of LV (anterior and anteroseptal) and SR to pre-infarcted level. In contrast, regional contractile function of untreated animals continued to decline after MI. These findings confirm our hypothesis that transplantation of hPSC-CMs improves regional myocardial strain within the infarct. Interestingly, the studies of Amado et al. [39] assessing changes in regional circumferential strain with HARP method in Yorkshire pigs after transplantation of allogeneic mesenchymal stem cells (MSC) are almost identical to our results. In their study, MSC-treated animals demonstrated an improvement in regional peak strain at the mid-wall region, from −4.6 ± 0.3% (at baseline) to −10.8 ± 0.6% (at 8 weeks post-MSC transplantation), whether no strain improvement was noted in infarcted area for duration of the study [39].

These findings support the use of our novel myocardial strain analysis to detect potential benefits of cardiac regenerative therapies.

The presence of a healthy hPSC-CM graft in the infarcted zones of the minipig’s heart supports the putative mechanism of regeneration of functional myocardium resulting in regional strain improvement. Our prior studies of hPSC-CM transplantation in NHPs also suggest that repopulation of cardiomyocytes in the infarcted zones underlies regional strain improvement [1], [2], [3]. Although the current study did not aim to define the mechanism of improved cardiac function, it supports the notion that either direct contractile or indirect paracrine benefits can be delivered by administering human cardiomyocytes to the infarct. The lack of improvement in global contractility (e.g., EF) may be related to a relative underdosing of cell therapy (limited by the increased risk of lethal engraftment arrhythmias in pig [5]), compared to the significantly larger recipient heart in pig [41] or greater species-species mismatch. Notably, Romagnuolo et al. also reported lack of efficacy when assessing global contractility for direct epicardial hESC-CM transplantation into pig [5]. With recent advances in eliminating engraftment arrhythmia [25], [26], it may be possible to do dose-escalation studies to assess whether global contractile function can be improved in the infarcted pig heart, but it does not exclude that other mechanisms might play a role in the global recovery. Also, *in vivo* graft maturation occurs slowly, taking upwards of three months to provide full functional benefit [42], [43], [44]. A longer follow-up time might be needed to prove therapeutic effect of hPSC-CM therapy of myocardial infarction. Moreover, it remains unclear whether either of those assessments would be predictive of positive clinical outcomes following cell therapies.

This study is proof of the concept for a novel method of myocardial strain analysis to (1) distinguish normal from infarcted myocardium and (2) detect subclinical improvements in regional contractile function after transendocardial hPSC-CM transplantation for myocardial infarction in minipigs.

We used myocardial tagging as the state-of-the-art CMR method for evaluating cardiac mechanics in response to treatment and a novel frequency-based approach for analysis of linear tags placed in 60-degree pattern offsets. This approach provided robust circumferential strain results sensitive for regional changes of circumferential strain following myocardial infarction and cell transplantation. Direct comparison of the new frequency-based approach with feature tracking method showed differences in absolute strain values but similarity of direction of circumferential strain changes over time. The feature tracking-based myocardial strain results suggested a similar conclusion; however, FT results had larger data variability and were highly dependent on image quality, while CSPAMM acquisition provided high-quality tagging cine images that can be easily analyzed with our novel approach based on localized Fourier transforms perpendicular to the applied tag pattern. The main advantages of the novel approach in comparison with FT are quick and robust algorithm for detection of regional myocardial deformation, absence of multiple pages hard-to-navigate results, and no cost.

## 5 Limitations

The current study has several limitations. Small sample size, relatively short follow-up observation period and underdosing of the cell product limit our ability to detect change in global contractile function. Strain analysis of only a single mid-ventricular short-axis slice might not be representative of the entire ventricle. Future studies are needed to understand the mechanism of improved CS in the absence of significantly improved ejection fraction. Further development of our method may include multislice acquisition of tagged images and slice addition in the long axis of the heart which would allow calculation of the longitudinal and radial strain.

## 6 Conclusion

A new method of myocardial tagging analysis has been developed and implemented for the first time to evaluate human cardiac cell therapy in the infarcted minipig heart. Our new method based on three orientations of tags at 60-degrees pattern aligned with AHA heart segments provide robust assessment of circumferential strain and strain rate in the infarcted minipig’s heart. A novel frequency-based technique for assessment of local circumferential strain does not require specialized acquisition protocols, access to k-space data, nor highly optimized reconstruction algorithms or commercial software. The novel approach successfully detected a decrease in circumferential strain after MI and recovery of myocardial strain followed human cardiomyocyte transplantation in comparison with untreated controls. The new myocardial strain assessment approach was validated against the standard tag tracking approach and feature tracking strain analysis and showed similar circumferential strain changes over time across the experimental conditions (normal heart, infarcted, cell/vehicle treated).

## Funding

These studies were supported by the UW Medicine Heart Regeneration Program, the Washington Research Foundation, a gift from Mike and Lynn Garvey, and a sponsored research agreement from Sana Biotechnology (all Seattle, WA). This work also was supported in part by NIH grants R01HL128368, R01HL146868, and R01HL148081 (to C.E.M.), a grant from the Fondation Leducq Transatlantic Network of Excellence (Boston, MA; to C.E.M.), the Bruce-Laughlin Research Fellowship (Seattle, WA; to K.N.), and the American Heart Association 24SCEFIA1253490 (to S.M.).

## Author contributions

AVN participated in data acquisition, image analysis, statistical analysis, interpretation of data, and manuscript writing. KN participated in data analysis and manuscript editing. LEN and LPB were responsible for animal procedures and surgery. HT and SM were responsible for cell preparation, histological analyses and manuscript editing (SM). WSK created a concept of strain analysis and software, participated in data interpretation and manuscript writing. CEM oversaw the study design and main concepts of the study. All authors read and approved of the final manuscript.

## Ethics approval and consent

Not applicable.

## Consent for publication

Not applicable.

## Declaration of competing interests

The authors declare the following financial interests/personal relationships which may be considered as potential competing interests: Charles E. Murry reports financial support was provided by Washington Research Foundation. Charles E. Murry reports financial support was provided by gift from Mike and Lynn Garvey. Charles E. Murry reports financial support was provided by Sana Biotechnology. Charles E. Murry reports financial support was provided by National Institutes of Health. Charles E. Murry reports financial support was provided by Fondation Leducq Transatlantic Network of Excellence. Kenta Nakamura reports financial support was provided by Bruce-Laughlin Research Fellowship. Silvia Marchiano reports financial support was provided by American Heart Association Inc. Charles E. Murry reports a relationship with Sana Biotechnology Inc that includes: equity or stocks. Charles E. Murry reports a relationship with StemCardia Inc that includes equity or stocks. If there are other authors, they declare that they have no known competing financial interests or personal relationships that could have appeared to influence the work reported in this paper.

## Data Availability

The dataset(s) supporting the conclusions of this article and the MATLAB software for tagging analysis are available upon request to authors.
