# Supplementary material for: Circumferential strain recovery after human cardiomyocyte transplantation in minipigs using a novel frequency-based method for myocardial tagging quantification
Source: J Cardiovasc Magn Reson. 2026 Jun 5;28(2):102756. doi: 10.1016/j.jocmr.2026.102756 (PMC13311266; doi:10.1016/j.jocmr.2026.102756)
Supplement: Supplementary file 1 — Supplementary material [file mmc1.docx]

Global and segmental circumferential end-systolic myocardial strain (CS) in normal minipig’s heart at the baseline before myocardial infarction assessed with the novel frequency-based technique and feature-tracking method.

|  | Novel frequency-based method | | | Feature-tracking (FT) method | | |
| --- | --- | --- | --- | --- | --- | --- |
| Measurement | Vehicle control group (n=5) | Cells group (n=4) | p-value differences between groups | Vehicle control group (n=5) | Cells group (n=4) | p-value differences between groups |
| Global peak CS, % | -6.36 ± 0.35 | -5.41 ± 1.84 | 0.3515 | -12.56 ± 2.96 | -15.28 ± 2.79 | 0.2815 |
| Anterior (A) CS, % | -5.50 ± 0.56 | -6.58 ± 2.16 | 0.3546 | -12.40 ±3.27 | -15.92 ±2.45 | 0.2265 |
| Anteroseptal (AS) CS, % | -4.86 ± 0.41 | -6.81 ± 2.12 | 0.2556 | -11.72 ± 1.68 | -13.84± 1.15 | 0.1980 |
| Inferoseptal (IS) CS, % | -5.68 ± 1.03 | -4.62 ± 1.38 | 0.3044 | -16.07 ± 2.83 | -14.39 ± 2.57 | 0.4006 |
| Inferior (I) CS, % | -6.27 ± 1.22 | -5.37 ± 2.24 | 0.3877 | -12.88 ± 2.51 | -8.66 ± 0.67 | 0.0965 |
| Inferolateral (IL) CS, % | -8.25 ± 0.64 | -7.67 ± 2.58 | 0.4325 | -12.44 ± 3.24 | -18.83 ±2.28 | 0.0932 |
| Anterolateral (AL) CS, % | -6.94 ±0.61 | -7.64 ± 1.15 | 0.3331 | -16.21 ± 3.15 | -17.57 ± 0.76 | 0.3510 |

Results are shown as mean ± standard error.

* marks statistically significant difference between vehicle and cell treated groups (p<0.05, t-test).

# marks statistically significant difference with baseline values of each studied group (p<0.05).

One tail p-values are shown.
