# Supplementary material for: Circumferential strain recovery after human cardiomyocyte transplantation in minipigs using a novel frequency-based method for myocardial tagging quantification
Source: J Cardiovasc Magn Reson. 2026 Jun 5;28(2):102756. doi: 10.1016/j.jocmr.2026.102756 (PMC13311266; doi:10.1016/j.jocmr.2026.102756)
Supplement: Supplementary file 2 — Supplementary material [file mmc10.pptx]

## Slide 1
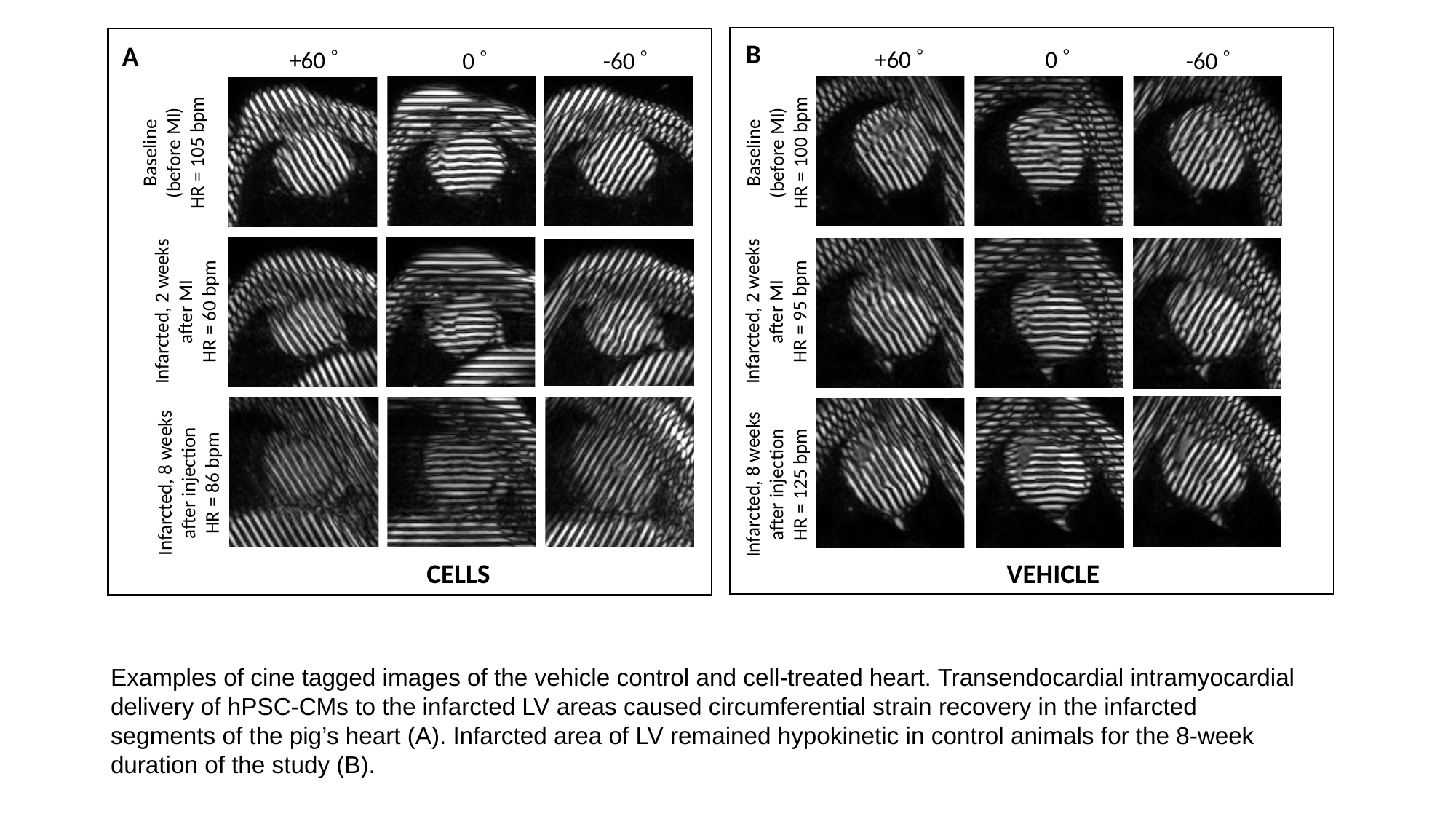

B
A
+60 
0 
+60 
0 
-60 
-60 
Baseline (before MI)
HR = 105 bpm
Baseline (before MI)
HR = 100 bpm
Infarcted, 2 weeks after MI
HR = 60 bpm
Infarcted, 2 weeks after MI
HR = 95 bpm
Infarcted, 8 weeks after injection
HR = 86 bpm
Infarcted, 8 weeks after injection
HR = 125 bpm
CELLS
VEHICLE
Examples of cine tagged images of the vehicle control and cell-treated heart. Transendocardial intramyocardial delivery of hPSC-CMs to the infarcted LV areas caused circumferential strain recovery in the infarcted segments of the pig’s heart (A). Infarcted area of LV remained hypokinetic in control animals for the 8-week duration of the study (B).
