# Supplementary figures and images for: Circumferential strain recovery after human cardiomyocyte transplantation in minipigs using a novel frequency-based method for myocardial tagging quantification
Source: J Cardiovasc Magn Reson. 2026 Jun 5;28(2):102756. doi: 10.1016/j.jocmr.2026.102756 (PMC13311266; doi:10.1016/j.jocmr.2026.102756)

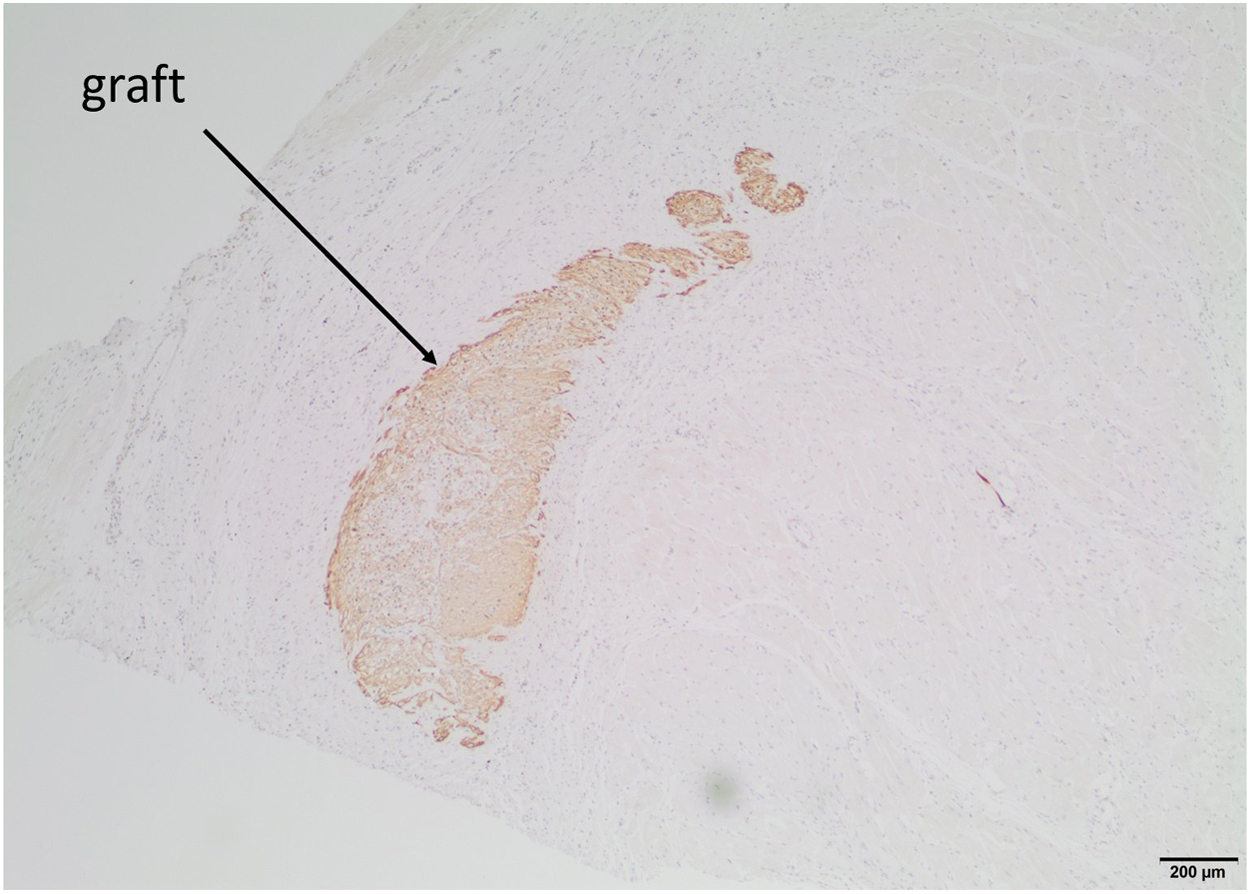

Supplement: Supplementary file 3 — Supplementary material [file mmc11.jpg]
