# Supplementary material for: Circumferential strain recovery after human cardiomyocyte transplantation in minipigs using a novel frequency-based method for myocardial tagging quantification
Source: J Cardiovasc Magn Reson. 2026 Jun 5;28(2):102756. doi: 10.1016/j.jocmr.2026.102756 (PMC13311266; doi:10.1016/j.jocmr.2026.102756)
Supplement: Supplementary file 4 — Supplementary material [file mmc2.docx]

Global and segmental circumferential end-systolic myocardial strain (CS) in minipig’s heart 2 weeks after myocardial infarction (MI) calculated with the novel frequency-based technique and feature-tracking method.

| Novel frequency-based method | | | | | | Feature-tracking method | | | | |
| --- | --- | --- | --- | --- | --- | --- | --- | --- | --- | --- |
| Measurements | Vehicle control group (n=5) | Cells group (n=4) | p-value differences between groups | p-value differences with the baseline (before MI) of the vehicle control group | p-value differences with the baseline (before MI) of the cell group | Vehicle control group (n=5) | Cells group (n=4) | p-value differences between groups | p-value differences with the baseline (before MI) of the vehicle control group | p-value differences with the baseline (before MI) of the cell group |
| Global peak CS, % | -6.08 ± 0.62 | -5.95 ± 0.66 | 0.4450 | 0.4070 | 0.4085 | -9.21 ± 0.61 | -11.58 ± 1.18 | 0.0707 | 0.1669 | 0.2772 |
| Anterior (A) CS, % | -3.27 ± 2.06 | -3.15 ± 3.00 | 0.4864 | 0.1047 | 0.4428 | -6.54 ± 3.54 | -3.41 ± 9.27 | 0.3842 | 0.3084 | 0.1619 |
| Anteroseptal (AS) CS, % | 0.48 ± 0.91 | 0.88 ± 2.50 | 0.4443 | 0.0148 # | 0.1645 | 1.76 ± 4.10 | 4.53 ± 3.44 | 0.3105 | 0.0479 # | 0.0092 # |
| Inferoseptal (IS) CS, % | -6.34 ± 0.86 | -7.11 ± 1.96 | 0.3694 | 0.4620 | 0.2333 | -14.18 ± 2.61 | -16.61 ± 4.75 | 0.3369 | 0.3315 | 0.4653 |
| Inferior (I) CS, % | -6.76 ± 2.27 | -5.36 ± 2.93 | 0.3592 | 0.2034 | 0.4690 | -9.19 ± 7.22 | -6.78 ± 3.95 | 0.3893 | 0.3153 | 0.2734 |
| Inferolateral (IL) CS, % | -10.27 ± 1.61 | -7.00 ± 2.61 | 0.1664 | 0.0207 # | 0.1867 | -15.10 ±1.75 | -11.95 ± 3.24 | 0.2221 | 0.1830 | 0.0911 |
| Anterolateral (AL) CS, % | -9.47 ± 1.15 | -8.56 ± 1.98 | 0.3541 | 0.1729 | 0.4085 | -9.21 ± 0.61 | -11.58 ± 1.18 | 0.0707 | 0.1669 | 0.2772 |

Results are shown as mean ± standard error.

* marks statistically significant difference between vehicle and cell treated groups (p<0.05, t-test).

# marks statistically significant difference with baseline values of each studied group (p<0.05).

One tail p-values are shown.
