# Supplementary material for: Circumferential strain recovery after human cardiomyocyte transplantation in minipigs using a novel frequency-based method for myocardial tagging quantification
Source: J Cardiovasc Magn Reson. 2026 Jun 5;28(2):102756. doi: 10.1016/j.jocmr.2026.102756 (PMC13311266; doi:10.1016/j.jocmr.2026.102756)
Supplement: Supplementary file 5 — Supplementary material [file mmc3.docx]

Global and segmental circumferential end-systolic myocardial strain (CS) in minipig’s heart at 4 weeks after cell or vehicle injection calculated with the novel frequency-based technique and feature-tracking method.

|  | Novel frequency-based method | | | | | Feature-tracking method | | | | |
| --- | --- | --- | --- | --- | --- | --- | --- | --- | --- | --- |
| Measurements | Vehicle control group (n=5) | Cells group (n=4) | p-value differences between groups | p-value differences with the baseline (before MI) of the vehicle control group | p-value differences with the baseline (before MI) of the cell group | Vehicle control group (n=5) | Cells group (n=4) | p-value differences between groups | p-value differences with the baseline (before MI) of the vehicle control group | p-value differences with the baseline (before MI) of the cell group |
| Global peak CS, % | -2.68 ± 0.60 | -4.01 ± 0.83 | 0.2232 | 0.0142 # | 0.2925 | -6.76 ± 3.47 | -6.41 ± 1.95 | 0.4698 | 0.1242 | 0.0078 |
| Anterior (A) CS, % | 0.64 ± 2.83 | -3.82 ± 1.41 | 0.1299 | 0.0531 | 0.1445 | 2.16 ± 5.11 | -8.67 ± 0.11 | 0.0507 | 0.0946 | 0.1849 |
| Anteroseptal (AS) CS, % | 5.77 ± 0.81 | -1.03 ± 0.19 | 0.0016 * | 0.0004 # | 0.0276 # | 8.17 ± 1.55 | 4.03 ± 0.71 | 0.0388 * | 0.0003 # | 0.0611 |
| Inferoseptal (IS) CS, % | 1.70 ± 2.78 | -2.58 ± 0.09 | 0.3842 | 0.0404 # | 0.1149 | -5.30 ± 4.82 | -7.47 ± 3.63 | 0.3892 | 0.1461 | 0.0930 |
| Inferior (I) CS, % | -4.25 ± 2.49 | -2.63 ± 1.74 | 0.3362 | 0.1871 | 0.3193 | 0.28 ± 6.12 | -7.94 ± 0.12 | 0.1250 | 0.0514 | 0.1480 |
| Inferolateral (IL) CS, % | -11.34 ± 1.25 | -10.68 ± 3.69 | 0.4599 | 0.0759 | 0.4029 | -15.89 ± 1.66 | -12.02 ± 1.84 | 0.1711 | 0.2917 | 0.1873 |

Results are shown as mean ± standard error.

* marks statistically significant difference between vehicle and cell treated groups (p<0.05, t-test).

# marks statistically significant difference with baseline values of each studied group (p<0.05).

One tail p-values are shown.
