# Supplementary material for: Circumferential strain recovery after human cardiomyocyte transplantation in minipigs using a novel frequency-based method for myocardial tagging quantification
Source: J Cardiovasc Magn Reson. 2026 Jun 5;28(2):102756. doi: 10.1016/j.jocmr.2026.102756 (PMC13311266; doi:10.1016/j.jocmr.2026.102756)
Supplement: Supplementary file 6 — Supplementary material [file mmc4.docx]

Global and segmental circumferential end-systolic myocardial strain (CS) in minipig’s heart at 8 weeks after cell or vehicle injection calculated with the novel frequency-based technique and feature-tracking method.

|  | Novel frequency-based method | | | | | Feature-tracking method | | | | |
| --- | --- | --- | --- | --- | --- | --- | --- | --- | --- | --- |
| Measurements | Vehicle control group (n=5) | Cells group (n=4) | p-value differences between groups | p-value differences with the baseline (before MI) of the vehicle control group | p-value differences with the baseline (before MI) of the cell group | Vehicle control group (n=5) | Cells group (n=4) | p-value differences between groups | p-value differences with the baseline (before MI) of the vehicle control group | p-value differences with the baseline (before MI) of the cell group |
| Global peak CS, % | -4.03 ± 0.76 | -5.50 ± 0.08 | 0.0629 | 0.0502 # | 0.1518 | -7.26 ± 1.98 | -7.81 ± 0.65 | 0.4025 | 0.0269 # | 0.1880 |
| Anterior (A) CS, % | -3.11 ± 2.30 | -4.84 ± 0.95 | 0.2607 | 0.2879 | 0.3339 | 0.43 ± 4.22 | -4.82 ± 5.45 | 0.2642 | 0.0061 # | 0.1895 |
| Anteroseptal (AS) CS, % | 3.76 ± 2.35 | -3.16 ± 0.86 | 0.0202 * | 0.0070 # | 0.2014 | 10.19 ±2.19 | -8.21 ± 1.25 | 0.0002 * | 0.0008 # | 0.0275 # |
| Inferoseptal (IS) CS, % | -2.32 ± 1.56 | -0.83 ± 2.20 | 0.3232 | 0.1385 | 0.4793 | -7.78 ± 3.38 | -11.64 ± 4.75 | 0.2927 | 0.1356 | 0.3034 |
| Inferior (I) CS, % | -8.32 ± 1.37 | -5.37 ± 1.08 | 0.0833 | 0.1257 | 0.1148 | -13.68 ± 0.98 | -10.26 ± 0.90 | 0.0308 * | 0.3168 | 0.2609 |
| Inferolateral (IL) CS, % | -11.23 ± 1.00 | -9.35 ± 1.57 | 0.2135 | 0.0029 # | 0.3335 | -11.98 ± 1.16 | -11.93 ± 0.71 | 0.4885 | 0.3621 | 0.0265 |

Results are shown as mean ± standard error.

* marks statistically significant difference between vehicle control and cell treated groups (p<0.05, t-test).

# marks statistically significant difference with baseline values of each studied group (p<0.05).

One tail p-values are shown.
