# Supplementary material for: Circumferential strain recovery after human cardiomyocyte transplantation in minipigs using a novel frequency-based method for myocardial tagging quantification
Source: J Cardiovasc Magn Reson. 2026 Jun 5;28(2):102756. doi: 10.1016/j.jocmr.2026.102756 (PMC13311266; doi:10.1016/j.jocmr.2026.102756)
Supplement: Supplementary file 7 — Supplementary material [file mmc5.docx]

Global and segmental circumferential end-systolic myocardial strain rate (SR) in normal minipig’s heart at the baseline before myocardial infarction assessed with the novel frequency-based technique and feature-tracking method.

|  | Novel frequency-based method, %/s | | | Feature-tracking (FT) method, s^-1^ | | |
| --- | --- | --- | --- | --- | --- | --- |
| Measurement | Vehicle control group (n=5) | Cells group (n=4) | p-value differences between groups | Vehicle control group (n=5) | Cells group (n=4) | p-value differences between groups |
| Global Peak SR | -35.07 ± 4.43 | -25.11 ± 5.77 | 0.1433 | -1.14 ± 0.06 | -1.35 ± 0.17 | 0.2021 |
| Anterior (A) SR | -35.73 ± 9.34 | -29.20 ± 7.98 | 0.3203 | -0.43 ± 1.18 | -1.52 ± 1.36 | 0.3031 |
| Anteroseptal (AS) SR | -23.84 ± 5.27 | -22.08 ± 4.32 | 0.4092 | -1.82 ± 0.48 | -1.58 ± 0.34 | 0.2269 |
| Inferoseptal (IS) SR | -31.90 ± 9.97 | -22.97 ± 9.34 | 0.2859 | -1.78 ± 0.37 | -1.86 ± 0.32 | 0.4120 |
| Inferior (I) SR | -29.42 ± 6.64 | -28.61 ± 6.65 | 0.4701 | -2.07 ± 0.27 | -0.92 ± 1.11 | 0.2328 |
| Inferolateral (IL) SR | -43.82 ± 7.60 | -17.67 ± 10.71 | 0.0785 | -0.94 ± 0.98 | 0.32 ± 1.62 | 0.2965 |
| Anterolateral (AL) SR | -45.72 ± 4.52 | -30.16 ± 8.54 | 0.1258 | -1.75 ± 0.27 | -0.85 ± 1.41 | 0.3203 |

Results are shown as mean ± standard error.

* marks statistically significant difference between vehicle and cell treated groups (p<0.05, t-test).

# marks statistically significant difference with baseline values of each studied group (p<0.05).

One tail p-values are shown.
