# Supplementary material for: Circumferential strain recovery after human cardiomyocyte transplantation in minipigs using a novel frequency-based method for myocardial tagging quantification
Source: J Cardiovasc Magn Reson. 2026 Jun 5;28(2):102756. doi: 10.1016/j.jocmr.2026.102756 (PMC13311266; doi:10.1016/j.jocmr.2026.102756)
Supplement: Supplementary file 8 — Supplementary material [file mmc6.docx]

Global and segmental systolic circumferential strain rate (SR) in minipig’s heart 2 weeks after myocardial infarction (MI) calculated with the novel frequency-based technique and feature-tracking method.

|  | Novel frequency-based method, %/s | | | | | Feature-tracking (FT) method, s^-1^ | | | | |
| --- | --- | --- | --- | --- | --- | --- | --- | --- | --- | --- |
| Measurements | Vehicle control group (n=5) | Cells group (n=4) | p-value differences between groups | p-value differences with the baseline (before MI) of the vehicle control group | p-value differences with the baseline (before MI) of the cell group | Vehicle control group (n=5) | Cells group (n=4) | p-value differences between groups | p-value differences with the baseline (before MI) of the vehicle control group | p-value differences with the baseline (before MI) of the cell group |
| Global Peak SR | -24.25 ± 3.12 | -17.23 ± 3.89 | 0.1037 | 0.0876 | 0.1833 | -0.66 ± 0.05 | -0.66 ± 0.06 | 0.4916 | 0.0149 | 0.0123 |
| Anterior (A) SR | -21.58 ± 10.7 | -1.47 ± 15.82 | 0.1682 | 0.0625 | 0.1902 | -0.56 ± 1.06 | 3.82 ± 4.61 | 0.2084 | 0.2907 | 0.3151 |
| Anteroseptal (AS) SR | 6.70 ± 6.34 | 16.17 ± 12.53 | 0.2666 | 0.0221 # | 0.0624 | 0.25 ± 0.98 | 4.72 ± 5.35 | 0.2341 | 0.1039 | 0.1282 |
| Inferoseptal (IS) SR | -18.48 ± 5.84 | -19.06 ± 8.57 | 0.4786 | 0.0755 | 0.4783 | -1.76 ± 0.56 | -0.32 ± 1.45 | 0.2029 | 0.4122 | 0.4168 |
| Inferior (I) SR | -23.65 ± 8.66 | -10.15 ± 9.66 | 0.1673 | 0.4275 | 0.2478 | -0.72 ± 1.08 | -2.12 ± 2.50 | 0.3172 | 0.1837 | 0.1004 |
| Inferolateral (IL) SR | -42.35 ± 8.01 | -21.66 ± 14.74 | 0.1375 | 0.2213 | 0.4399 | -1.88 ± 0.27 | -1.10 ± 0.61 | 0.1529 | 0.2174 | 0.3399 |
| Anterolateral (AL) SR | -46.11 ± 7.07 | -22.22 ± 6.68 | 0.0219 * | 0.3280 | 0.3619 | -0.30 ± 0.69 | -3.93 ± 2.63 | 0.1316 | 0.1203 | 0.3857 |

Results are shown as mean ± standard error.

* marks statistically significant difference between vehicle and cell treated groups (p<0.05, t-test).

# marks statistically significant difference with baseline values of each studied group (p<0.05).

One tail p-values are shown.
