# Supplementary material for: Circumferential strain recovery after human cardiomyocyte transplantation in minipigs using a novel frequency-based method for myocardial tagging quantification
Source: J Cardiovasc Magn Reson. 2026 Jun 5;28(2):102756. doi: 10.1016/j.jocmr.2026.102756 (PMC13311266; doi:10.1016/j.jocmr.2026.102756)
Supplement: Supplementary file 9 — Supplementary material [file mmc7.docx]

Global and segmental circumferential end-systolic myocardial strain rate (SR) in minipig’s heart at 4 weeks after cell or vehicle injection calculated with the novel frequency-based technique and feature-tracking method.

|  | Novel frequency-based method, %/s | | | | | Feature-tracking (FT) method, s^-1^ | | | | |
| --- | --- | --- | --- | --- | --- | --- | --- | --- | --- | --- |
| Measurements | Vehicle control group (n=5) | Cells group (n=4) | p-value differences between groups | p-value differences with the baseline (before MI) of the vehicle control group | p-value differences with the baseline (before MI) of the cell group | Vehicle control group (n=5) | Cells group (n=4) | p-value differences between groups | p-value differences with the baseline (before MI) of the vehicle control group | p-value differences with the baseline (before MI) of the cell group |
| Global Peak SR | -10.20 ± 7.60 | -7.56 ± 6.74 | 0.4226 | 0.02479 # | 0.0191 # | -0.13 ± 0.41 | -0.42 ±0.12 | 0.2789 | 0.0383 # | 0.0385 # |
| Anterior (A) SR | 0.45 ± 10.46 | -21.17 ± 0.38 | 0.0538 | 0.0523 | 0.3304 | 1.72 ± 0.81 | -1.19 ± 0.06 | 0.0117 * | 0.0458 | 0.3658 |
| Anteroseptal (AS) SR | 16.09 ± 6.25 | 26.38 ± 8.88 | 0.2795 | 0.0098 # | 0.0767 | -0.47 ± 0.94 | -0.31 ± 0.57 | 0.4508 | 0.2573 | 0.3482 |
| Inferoseptal (IS) SR | 1.09 ± 8.82 | 14.55 ± 1.35 | 0.1022 | 0.0346 # | 0.1217 | -1.24 ± 0.97 | -0.72 ± 0.06 | 0.3091 | 0.4939 | 0.2393 |
| Inferior (I) SR | -19.54 ± 11.85 | -11.58 ± 11.26 | 0.3617 | 0.1886 | 0.0666 | -0.47 ± 1.01 | 0.28 ± 0.60 | 0.2999 | 0.1353 | 0.4738 |
| Inferolateral (IL) SR | -40.35 ± 11.37 | -38.39 ± 11.32 | 0.4645 | 0.2424 | 0.1658 | -2.58 ± 0.76 | -1.22 ± 0.14 | 0.0750 | 0.1650 | 0.2824 |
| Anterolateral (AL) SR | -18.97 ± 11.30 | -15.15 ± 7.25 | 0.4081 | 0.0029 # | 0.1459 | 0.02 ± 1.10 | -1.22 ± 0.13 | 0.1631 | 0.1333 | 0.2949 |

Results are shown as mean ± standard error.

* marks statistically significant difference between vehicle and cell treated groups (p<0.05, t-test).

# marks statistically significant difference with baseline values of each studied group (p<0.05).

One tail p-values are shown.
