# Supplementary material for: Circumferential strain recovery after human cardiomyocyte transplantation in minipigs using a novel frequency-based method for myocardial tagging quantification
Source: J Cardiovasc Magn Reson. 2026 Jun 5;28(2):102756. doi: 10.1016/j.jocmr.2026.102756 (PMC13311266; doi:10.1016/j.jocmr.2026.102756)
Supplement: Supplementary file 10 — Supplementary material [file mmc8.docx]

Global and segmental circumferential end-systolic myocardial strain rate (SR) in minipig’s heart at 8 weeks after cell or vehicle injection calculated with the novel frequency-based technique and feature-tracking method.

|  | Novel frequency-based method, %/s | | | | | Feature-tracking (FT) method, s^-1^ | | | | |
| --- | --- | --- | --- | --- | --- | --- | --- | --- | --- | --- |
| Measurements | Vehicle control group (n=5) | Cells group (n=4) | p-value differences between groups | p-value differences with the baseline (before MI) of the vehicle control group | p-value differences with the baseline (before MI) of the cell group | Vehicle control group (n=5) | Cells group (n=4) | p-value differences between groups | p-value differences with the baseline (before MI) of the vehicle control group | p-value differences with the baseline (before MI) of the cell group |
| Global Peak SR | -17.75 ± 3.67 | -17.88 ± 3.43 | 0.4908 | 0.0052 # | 0.1593 | -0.44 ± 0.34 | -0.24 ± 0.22 | 0.3227 | 0.0878 | 0.4919 # |
| Anterior (A) SR | -14.17 ± 7.32 | -20.22 ± 8.81 | 0.3271 | 0.0744 | 0.2626 | -0.4 ± 0.95 | -0.49 ± 0.75 | 0.4717 | 0.4723 | 0.4820 |
| Anteroseptal (AS) SR | 14.36 ± 10.54 | -20.11 ± 6.39 | 0.0183 * | 0.0088 # | 0.1150 | 0.79 ± 1.16 | -0.42 ± 0.59 | 0.2014 | 0.1330 | 0.0732 |
| Inferoseptal (IS) SR | -8.83 ± 8.18 | -4.86 ± 3.69 | 0.3415 | 0.1699 | 0.2154 | -2.08 ± 0.98 | -0.96 ± 1.45 | 0.3000 | 0.1129 | 0.4799 |
| Inferior (I) SR | -26.47 ± 7.82 | -4.71 ± 7.80 | 0.0651 | 0.3321 | 0.1787 | -1.70 ± 0.49 | -1.28 ± 0.16 | 0.2278 | 0.4159 | 0.2964 |
| Inferolateral (IL) SR | -43.42 ± 7.71 | -21.68 ± 7.69 | 0.0631 | 0.4731 | 0.3861 | -1.96 ± 0.45 | -0.80 ± 1.38 | 0.2737 | 0.2142 | 0.2646 |
| Anterolateral (AL) SR | -27.95 ± 7.36 | -35.69 ± 5.73 | 0.2330 | 0.0275 # | 0.1344 | -0.41 ± 1.27 | -2.70 ± 1.56 | 0.4912 | 0.1780 | 0.3547 |

Results are shown as mean ± standard error.

* marks statistically significant difference between vehicle and cell treated groups (p<0.05, t-test).

# marks statistically significant difference with baseline values of each studied group (p<0.05).

One tail p-values are shown.
