# Supplementary material for: Circumferential strain recovery after human cardiomyocyte transplantation in minipigs using a novel frequency-based method for myocardial tagging quantification
Source: J Cardiovasc Magn Reson. 2026 Jun 5;28(2):102756. doi: 10.1016/j.jocmr.2026.102756 (PMC13311266; doi:10.1016/j.jocmr.2026.102756)
Supplement: Supplementary file 11 — Supplementary material [file mmc9.docx]

Parameters of the global contractile function of the minipig’s heart in the vehicle control group and the group treated with human cardiomyocytes at the baseline before myocardial infarction (MI), 2 weeks after MI, at 4 and 8 weeks after cell or vehicle injection.

| **Baseline (before MI)** | | | | | |
| --- | --- | --- | --- | --- | --- |
| Measurement | Vehicle control (n=5) | Cell treated  (n=4) | p-value differences between groups | p-value differences with baseline of the vehicle control group | p-value differences with baseline of the cell treated group |
| HR, bpm | 87 ± 11 | 70 ± 16 | 0.2365 |  |  |
| ESV, ml | 12.8 ± 1.5 | 10.6 ± 0.9 | 0.1453 |  |  |
| EDV, ml | 29.4 ± 3.4 | 24.8 ± 2.6 | 0.1761 |  |  |
| SV, ml | 16.6 ± 2.0 | 14.2 ± 1.8 | 0.2148 |  |  |
| LV mass, g | 42.4 ± 2.1 | 45.5 ± 4.6 | 0.3167 |  |  |
| CO, liter/min | 1.5 ± 0.1 | 0.9 ± 0.2 | 0.0681 |  |  |
| EF, % | 56 ± 1.2 | 57 ± 1.3 | 0.3860 |  |  |
| **2 weeks after MI** | | | | | |
| Measurement | Vehicle control (n=5) | Cell treated  (n=4) | p-value differences between groups | p-value differences with baseline of the vehicle control group | p-value differences with baseline of the cell treated group |
| HR, bpm | 84 ± 7 | 65 ± 5 | 0.03159 * | 0.21742 | 0.365819 |
| ESV, ml | 24.1 ± 1.1 | 26.7 ± 3.9 | 0.2417 | 0.000455 # | 0.032448 # |
| EDV, ml | 39.4 ± 0.9 | 44.7 ± 6.8 | 0.2466 | 0.013843 # | 0.056549 |
| SV, ml | 15.2 ± 1.3 | 18.0 ± 3.1 | 0.2349 | 0.268629 | 0.144748 |
| LV mass, g | 55 ± 3.2 | 64.7 ± 5.8 | 0.1020 | 0.012923 # | 0.004329 # |
| CO, liter/min | 1.3 ± 0.2 | 1.2 ± 0.2 | 0.3353 | 0.090652 | 0.091752 |
| EF, % | 39 ± 3 | 40 ± 2.3 | 0.4009 | 0.000473 # | 0.013726 # |
| **4 weeks after cell/vehicle delivery** | | | | | |
| Measurement | Vehicle control (n=5) | Cell treated  (n=4) | p-value differences between groups | p-value differences with baseline of the vehicle control group | p-value differences with baseline of the cell treated group |
| HR, bpm | 86 ± 7 | 76 ± 3 | 0.1657 | 0.031171 # | 0.039583 # |
| ESV, ml | 27.4 ± 3.4 | 40.0 ± 2.0 | 0.02423 * | 0.002052 # | 0.049958 # |
| EDV, ml | 42.8 ± 3.8 | 56.9 ± 1.2 | 0.2906 | 0.002129 # | 0.066991 |
| SV, ml | 15.4 ± 2.0 | 11.9 ± 2.7 | 0.2482 | 0.317146 | 0.398894 |
| LV mass, g | 65.7 ± 6.8 | 64.1 ± 2.8 | 0.4232 | 0.006361 # | 0.122793 |
| CO, liter/min | 1.3 ± 0.2 | 0.9 ± 0.2 | 0.1908 | 0.169153 | 0.415249 |
| EF, % | 36 ± 4 | 31 ± 3 | 0.2197 | 0.002226 # | 0.023536 # |
| **8 weeks after cell/vehicle delivery** | | | | | |
| Measurement | Vehicle control (n=5) | Cell treated  (n=4) | p-value differences between groups | p-value differences with baseline of the vehicle control group | p-value differences with baseline of the cell treated group |
| HR, bpm | 94 ± 9 | 80 ± 4 | 0.1058 | 0.461765 | 0.397584 |
| ESV, ml | 30.3 ± 2.7 | 28.3 ± 6.1 | 0.4057 | 0.000746 # | 0.079635 |
| EDV, ml | 50.4 ± 3.2 | 48.0 ± 7.0 | 0.4033 | 0.000934 # | 0.032426 # |
| SV, ml | 20.0 ± 3.1 | 19.7 ± 2.6 | 0.4701 | 0.159487 | 0.09813 |
| LV mass, g | 71.1 ± 7.9 | 60.7 ± 2.5 | 0.1344 | 0.006234 # | 0.133446 |
| CO, liter/min | 1.8 ± 0.2 | 1.6 ± 0.3 | 0.3638 | 0.091113 | 0.031726 # |
| EF, % | 40 ± 5 | 42 ± 6 | 0.3813 | 0.008525 # | 0.147584 |

Data is shown as mean ± standard error of the mean.

* marks statistically significant difference with vehicle (control) cohort at the same time point (p<0.05).

# marks statistically significant difference with baseline values of each studied group (p<0.05).

One tail p-values are shown.
